# Supplementary material for: Pilot study to establish a prospective neonatal cohort: Study of Preterm Infants and Neurodevelopmental Genes (SPRING)
Source: BMJ Paediatr Open. 2020 Jul 30;4(1):e000648. doi: 10.1136/bmjpo-2020-000648 (PMC7394180; doi:10.1136/bmjpo-2020-000648)
Supplement: Supplementary data [file bmjpo-2020-000648supp001.pdf]

Supplementary figure. Geographical distribution of participating neonatal units (each represented by a marker) shown on a map of England, United Kingdom, 2017 – 2018

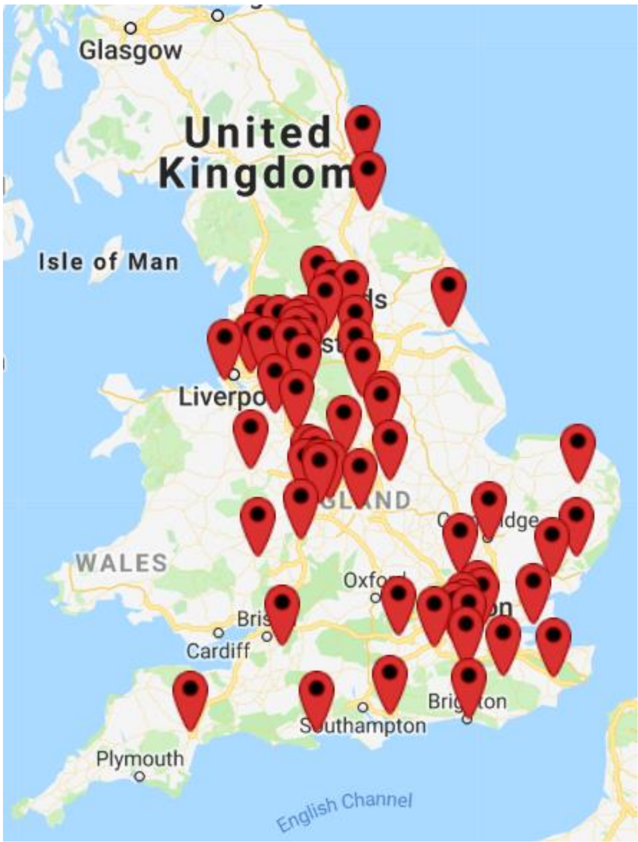

<u>List of participating hospitals</u>:

Airedale General Hospital
Arrowe Park Hospital
Barnsley District General Hospital
Birmingham City Hospital
Birmingham Heartlands Hospital
Birmingham Women's Hospital
Bradford Royal Infirmary
Calderdale Royal Hospital
Chelsea & Westminster Hospital
Chesterfield & North Derbyshire Royal Hospital
Colchester General Hospital
Croydon University Hospital
East Surrey Hospital
Hereford County Hospital
Hull Royal Infirmary
Ipswich Hospital
Kingston Hospital
Leeds Neonatal Service
Leicester General Hospital

Lister Hospital
Macclesfield District General Hospital
Manor Hospital
New Cross Hospital
Newham Hospital
Norfolk & Norwich University Hospital
North Manchester
North Tyneside General Hospital
Nottingham City Hospital/
Poole General Hospital
Queen Alexandra Hospital (Portsmouth)
Queen's Hospital, Burton on Trent
Queen's Medical Centre, Nottingham University Hospital
Rosie Maternity Hospital, Addenbrookes
Royal Albert Edward Infirmary
Royal Berkshire Hospital
Royal Bolton Hospital
Royal Devon & Exeter Hospital
Royal Oldham Hospital
Royal Shrewsbury Hospital
Royal Stoke University Hospital
Royal Sussex County Hospital
Royal United Hospital, Bath
Russells Hall Hospital
Southend Hospital
St George's Hospital
St Mary's Hospital, Manchester
St Peter's Hospital
Stepping Hill Hospital
Sunderland Royal Hospital
Tameside General Hospital
The Jessop Wing, Sheffield
Tunbridge Wells Hospital
University Hospital Coventry
University Hospital of North Tees
University Hospital of South Manchester
Warrington Hospital
Whipps Cross University Hospital/
Whiston Hospital
William Harvey Hospital
Worcestershire Royal Hospital
