# Supplementary material for: Pilot study to establish a prospective neonatal cohort: Study of Preterm Infants and Neurodevelopmental Genes (SPRING)
Source: BMJ Paediatr Open. 2020 Jul 30;4(1):e000648. doi: 10.1136/bmjpo-2020-000648 (PMC7394180; doi:10.1136/bmjpo-2020-000648)
Supplement: Author's manuscript [file bmjpo-2020-000648.draft_revisions.pdf]

BMJ Paediatrics Open

BMJ Paediatrics Open is committed to open peer review. As part of this commitment we make the peer review history of every article we publish publicly available.

When an article is published we post the peer reviewers' comments and the authors' responses online. We also post the versions of the paper that were used during peer review. These are the versions that the peer review comments apply to.

The versions of the paper that follow are the versions that were submitted during the peer review process. They are not the versions of record or the final published versions. They should not be cited or distributed as the published version of this manuscript.

BMJ Paediatrics Open is an open access journal and the full, final, typeset and author-corrected version of record of the manuscript is available on our site with no access controls, subscription charges or pay-per-view fees (http://bmjpaedsopen.bmj.com).

If you have any questions on BMJ Paediatrics Open's open peer review process please email info.bmjpo@bmj.com

BMJ Paediatrics Open

Establishing a prospective neonatal cohort for clinical and genetic investigations: experiences of the pilot Study of Preterm Infants and Neurodevelopmental Genes (SPRING)

Journal:	*BMJ Paediatrics Open*
Manuscript ID	bmjpo-2020-000648
Article Type:	Original research
Date Submitted by the Author:	30-Jan-2020
Complete List of Authors:	Wong, Hilary; University of Cambridge School of Clinical Medicine, Department of Paediatrics<br>Hopkins, Lucinda; Cardiff University, MRC Centre for Neuropsychiatric Genetics and Genomics<br>O'Donovan, Michael; Cardiff University, MRC Centre for Neuropsychiatric Genetics and Genomics<br>Thapar, Anita; Cardiff University, MRC Centre for Neuropsychiatric Genetics and Genomics<br>Modi, Neena; Imperial College London Department of Medicine, Section of Neonatal Medicine
Keywords:	Autism, Epidemiology, Genetics, Neonatology, Neurodevelopment

SCHOLARONE™
Manuscripts

I, the Submitting Author has the right to grant and does grant on behalf of all authors of the Work (as defined in the below author licence), an exclusive licence and/or a non-exclusive licence for contributions from authors who are: i) UK Crown employees; ii) where BMJ has agreed a CC-BY licence shall apply, and/or iii) in accordance with the terms applicable for US Federal Government officers or employees acting as part of their official duties; on a worldwide, perpetual, irrevocable, royalty-free basis to BMJ Publishing Group Ltd ("BMJ") its licensees and where the relevant Journal is co-owned by BMJ to the co-owners of the Journal, to publish the Work in this journal and any other BMJ products and to exploit all rights, as set out in our licence.

The Submitting Author accepts and understands that any supply made under these terms is made by BMJ to the Submitting Author unless you are acting as an employee on behalf of your employer or a postgraduate student of an affiliated institution which is paying any applicable article publishing charge ("APC") for Open Access articles. Where the Submitting Author wishes to make the Work available on an Open Access basis (and intends to pay the relevant APC), the terms of reuse of such Open Access shall be governed by a Creative Commons licence – details of these licences and which Creative Commons licence will apply to this Work are set out in our licence referred to above.

Other than as permitted in any relevant BMJ Author's Self Archiving Policies, I confirm this Work has not been accepted for publication elsewhere, is not being considered for publication elsewhere and does not duplicate material already published. I confirm all authors consent to publication of this Work and authorise the granting of this licence.

Confidential: For Review Only

Establishing a prospective neonatal cohort for clinical and genetic investigations: experiences of the pilot Study of Preterm Infants and Neurodevelopmental Genes (SPRING)

Authors:

Hilary S Wong[1]

Lucinda Hopkins[2]

Michael C O'Donovan[2]

Anita Thapar[2]

Neena Modi[3]

Affiliations:

1. Department of Paediatrics, University of Cambridge, Cambridge, United Kingdom
2. MRC Centre for Neuropsychiatric Genetics and Genomics, Cardiff University School of Medicine, Cardiff, United Kingdom
3. Section of Neonatal Medicine, Chelsea and Westminster campus, Imperial College London, London, United Kingdom

Corresponding author:

Professor Neena Modi
Section of Neonatal Medicine
Imperial College London
Chelsea and Westminster campus
369 Fulham Road
London SW10 9NH
Email: n.modi@imperial.ac.uk
Telephone: 020 3315 5101

Word count: 2520 words

Confidential: For Review Only

ABSTRACT

Background: Genetic risk variants and preterm birth are early and potent risk factors for later neuropsychiatric disorders. To understand the interrelationships between these factors, a large-scale genetic study of very preterm (VPT, <32 weeks gestation) infants with prospective follow-up is required. In this paper we describe a streamlined study approach, using efficient processes for biological and clinical data collection, to feasibly establish such a cohort.

Methods: We sought to recruit 500 VPT families within a one-year period from neonatal units. Treating clinical teams recruited eligible participants, obtained parent consent, collected blood samples and posted specimens to the research laboratory. We extracted all clinical data from the National Neonatal Research Database, an existing UK resource that captures daily patient-level data on all VPT infants.

Results: Between May 2017 and June 2018, we established a cohort of 848 VPT infants and their parents from 60 English neonatal units. The study population (median (inter-quartile range) gestation 28.9 (26-30) weeks; birthweight 1120 (886-1420) grams) represented 18.9% of eligible infants born at the study sites during the recruitment period (n= 4491). From the subset of 521 complete family trios, we successfully completed genotyping for 510 (97.9%) trios. Of the original 883 infants whose parents consented to participate, the parents of 796 (90.1%) infants agreed to future data linkage and 794 (89.9%) agreed to be recalled.

Conclusion: We demonstrate the feasibility and acceptability of streamlined strategies for genetic, neonatal and longitudinal data collection and provide a template for future cost-effective and efficient cohort development.

(248 of max 250 words)

Keywords: Preterm birth, prospective cohort study, neurodevelopment, autism spectrum disorder, attention deficit hyperactivity disorder, genetics, epidemiology, data linkage

Confidential: For Review Only

Confidential: For Review Only

BACKGROUND

Globally, approximately 15 million infants are born preterm (<37 weeks gestation) each year and the prevalence is rising [1]. The causes of spontaneous preterm births and the conditions justifying 'medically-indicated' preterm birth are complex and poorly understood, with both environmental factors [2,3] and genetic components [4] implicated. Preterm infants experience significant adversities and are at elevated risk of intellectual disability, behavioural and mental health problems, with approximately a quarter of children born extremely preterm meeting neuropsychiatric diagnostic criteria by childhood [5]. However, there is considerable heterogeneity in outcomes, the causes of which are unknown.

Previous epidemiological studies have suggested family and genetic factors may contribute to the link between preterm birth and later neuropsychiatric disorders. Many of these studies have observed an association between maternal mental health problems and risk of preterm birth [6,7]. Positive family history of psychiatric disorder further contributes to the risk of adverse mental health outcomes among individuals born preterm [8,9]. Whilst genomic studies have identified large numbers of genetic risk variants for major neuropsychiatric disorders, including those influencing risks for attention deficit hyperactivity disorder and autism spectrum disorder, the neuropsychiatric disorders most strongly associated with preterm birth in childhood [10], the relevance of these genetic risk variants for those born preterm has not been investigated.

A large preterm birth cohort with genomic data and prospective clinical phenotyping would be required to investigate the relationship between genetic risks, preterm birth and neuropsychiatric outcomes. There is no sufficiently powered existing prospective cohort of preterm infants for such investigations and the costs, time and resource requirements for recruitment and data collection by established methods are likely to be prohibitive. Our aims were to examine the feasibility of large-scale recruitment and DNA collection in a multi-centre setting, by capitalising on existing research infrastructure in the UK to streamline operational efficiencies, and the acceptability to parents of prospective follow-up through

routine data linkage and direct recall for detailed phenotyping. Our secondary aim was to conduct a preliminary investigation to explore the hypothesis that the very preterm (VPT, <32 weeks gestation) population is enriched for rare pathogenic copy number variants; the results of this investigation will be reported in a separate paper.

METHODS

Study sites and recruitment

We invited expressions of interest in participation as study sites from all 43 neonatal intensive care units (NICU) and 77 local neonatal units (LNU) in England. Of those that expressed interest, we selected study sites based on the potential number of eligible participants, geographical coverage and record of accomplishment of research activities in the unit. In the UK, neonatal care is provided within managed clinical networks where NICU provide tertiary specialist and long-term intensive care and LNU provide the full range of care for babies delivered in the nominal catchment area of the hospital [11]. The study was included in the National Institute for Health Research Clinical Research Network Portfolio, which allowed us to access support, such as CRN-funded research nurses in England. Being a 'portfolio study' also acts as an incentive for recruitment activities from neonatal units as the accrual data are used to inform future allocations of NHS research support funding to hospital trusts. All study procedures were carried out by the treating clinical or research staff based locally at the participating study sites. These local staff received training in the study procedures from the investigator team using training videos and teleconferences.

Recruitment took place between 1 May 2017 and 30 June 2018. Eligible participants were infants born VPT and their parents. Whilst we aimed to recruit both parents for trio-based genome analysis, single-parent families were also included. Similarly, we recruited infants where one or both parents declined to participate in the study themselves. We did not recruit parents known to be genetically unrelated to the

Confidential: For Review Only

child, for instance if the pregnancy had arisen from a donor gamete. Potential participants could be approached and recruited at any point during their neonatal hospitalisation at the discretion of the local research staff. Informed written consent was obtained from each parent (for themselves) and their offspring at the local site. We sought to recruit 500 VPT infants over the one-year recruitment phase.

Biological specimen collection and laboratory processing

Blood samples from participants were collected into EDTA specimen bottles. For infants, 0.5 to 1ml of blood were collected by heel lance, venepuncture or from an indwelling arterial catheter during blood sampling for routine clinical care to avoid an additional intervention for the infant. If the target volume was not achieved, a second aliquot was obtained, also collected during routine sampling. Five ml of blood was collected from each parent. The blood specimens were labelled using barcodes and a unique participant study number, packaged in a designated mailing box for biological specimens, in accordance with UN3373 regulations and P650 packaging instructions, and posted to the research laboratory at the MRC Centre for Neuropsychiatric Genetics and Genomics (MRC CNGG), Cardiff University within 7 days of sample collection using UK standard postal services. No laboratory processing of the samples took place at the recruitment study sites.

DNA was extracted from the blood specimens in-house at the MRC CNGG, from full sets of parent-infant trios in the first instance, using GE Healthcare DNA extraction kits. DNA sample quantification was determined using Quant-iT PicoGreen dsDNA assay kits and samples were genotyped using Illumina OmniExpress BeadChip arrays.

Clinical data collection

To minimise research workload and participant burden, we capitalised on the existing National Neonatal Research Database (NNRD) to access relevant clinical data on the research participants. Investigator NM led the establishment of the NNRD in 2007 to support the use of clinical data to improve neonatal care and outcomes, and the UK Neonatal Collaborative comprising all neonatal units that contributed data.

Confidential: For Review Only

Patient-level demographic and clinical point-of-care electronic data entered by treating clinicians on all infants admitted to the neonatal units are released with permission from the Caldicott Guardians to the Neonatal Data Analysis Unit at Imperial College London, where they are merged and cleaned to create the NNRD (https://www.imperial.ac.uk/neonatal-data-analysis-unit). The NNRD has complete population coverage of all neonatal unit admissions, including all VPT births in England, Wales and Scotland (approximately 7000 annually) and is approved by the UK National Research Ethics Service (10/80803/151) as a research database and by the Health Research Authority Confidentiality Advisory Group (8-05(f)/0210).

Upon receipt of parent signed authorisation, the unique NNRD identifier for each participating infant was obtained from his/her neonatologist. All relevant participant clinical data, including gestational age, birth weight, sex, time of onset of labour and maternal postcode, was extracted from the NNRD.

Consent for follow-up

Permissions were sought from parents of participating infants to conduct follow-up studies by means of linkage of the genetic and clinical data to future health and educational records. This was indicated by their response to the following question in the consent form: "*I give permission for the research team to link the information on my baby as part of the study to records that the NHS and other public organisations hold*". Consent was also sought for re-contacting the family in the future for detailed phenotypic assessments.

Research ethics

This study was reviewed and approved by the Heath Research Authority and the National Health Service Research Ethics Committee (reference 16/WA/0324).

Patient involvement

https://mc.manuscriptcentral.com/bmjpo

Confidential: For Review Only

We involved Bliss, the leading national preterm charity in the design and funding application for the study. We consulted parent representatives in developing the study materials including the participant information sheet. They will be consulted for the public dissemination of the findings from this research.

RESULTS

Recruitment and establishment of study population

We received expressions of interest from 75 out of 120 neonatal units in England. Of these, we selected 60 neonatal units (24 NICU and 36 LNU) to be study sites. These provided an extensive population catchment coverage in England (see supplementary material for geographical coverage and list of participating hospitals).

During the 14-month recruitment period, 1853 families were approached and 779 (42.0%) families, consisting of 883 infants, consented to participation (figure 1). Blood samples were not collected in 9 infants prior to their discharge. In 5 cases, the sample was lost during postage. Blood samples from 869 infants, 753 mothers and 551 fathers were successfully received in the MRC CNGG laboratory. Of these samples, there were 529 full infant-parent trios, quads (in twin births) or quints (in triplet births).

Clinical data extraction from the NNRD was successful in 848 (97.6%) infants whose blood samples had arrived at the laboratory. The demographic and neonatal characteristics of these participants are summarised in table 1.

During the recruitment period, a total of 8998 infants were born at <32 weeks gestation in England, with 4491 of whom being born at participating study sites. Therefore, this study cohort represent 18.9% of eligible infants born at study sites and 9.4% of the national VPT population. Our study sample was representative of the national VPT population in gestational age, birth weight, proportion of boys, singleton pregnancies, spontaneous onset of preterm labour and mode of delivery (table 1).

https://mc.manuscriptcentral.com/bmjpo

Confidential: For Review Only

Table 1 Neonatal characteristics of SPRING participants and of all VPT infants born in the participating study sites and in England during the recruitment period (May 2017 - June 2018)

	**SPRING participants** (n=848)	**All eligible infants born in study sites** (n=4491)	**National cohort** (n=8998)
Gestational age in weeks median (IQR)	28.9 (26 – 30)	29 (27 – 30)	29 (27 – 30)
Birth weight in grams median (IQR)	1120 (886 – 1420)	1175 (882 – 1480)	1170 (880 – 1470)
Boys, n (%)	456 (53.8)	2469 (55.0)	4900 (54.5)
Singleton pregnancy, n (%)	638 (75.2)	3359 (74.8)	6706 (74.5)
Spontaneous onset of labour, n (%)	474 (55.9)	2431 (54.1)	4815 (53.5)
Caesearean section delivery, n (%)	498 (58.7)	2584 (57.5)	5087 (56.5)
Maternal age at birth in years, Median (IQR)	31 (27-35)	Information not available	Information not available

DNA extraction and quality

DNA was extracted from a subset of blood samples from 521 full parent-infant trios who had successful linkage to infant clinical data. Of these, nine trios were excluded from genotyping due to inadequate DNA sample concentration of one of the trio members. An additional two trios were excluded due to array capacity, resulting in 510 trios (97.9%) of this subset being successfully genotyped.

Follow-up

Of the 883 infants whose parents consented to participate in the study, 796 (90.1%) agreed for their child's future health and educational data to be linked to the research data. Consent was also obtained from the parents of 794 (89.9%) for future direct contact.

DISCUSSION

Large-scale prospective cohort studies are time-consuming and expensive to conduct. Cohorts of special patient or high-risk groups, such as preterm infants, require assembly from multiple centres across large geographical areas, which poses additional logistical challenge. In our pilot study, we overcame some of

https://mc.manuscriptcentral.com/bmjpo

Confidential: For Review Only

these barriers by optimising research operational efficiencies. Although other large-scale preterm neonatal cohorts with genetic data exist internationally [12–16], none have collected parent DNA samples for trio-based genetic analyses. Parent blood samples for trio-based analysis is essential to identify likely pathogenic *de novo* variants. Our study approaches were purposefully designed to reduce research burden, respectful of the needs of preterm neonates and their families and sensitive to the culture within neonatal units. Within one year, we successfully established a large contemporaneous VPT neonatal cohort for genetic investigations.

Choice of recruitment and data collection methods

The completeness and quality of data held in the NNRD has been validated for use in research [17]. The advantages of leveraging routine electronic health records for research are well-recognised. These include less intrusive data collection, elimination of the need for duplicate data entry, and reduced research burden and cost. The reduced demands allowed us to incorporate the study activities within the workload of existing clinical and research staff at the participating centres, thereby minimising research costs.

As no research-specific prospective data collection was required, our study design also removed the time pressure to recruit newborn infants immediately after birth, particularly as this may be a stressful and emotional time for parents. Time constraints upon decision-making reduces willingness to participate [18]. Participants in our study were usually recruited a few weeks after delivery, when the parents had become more familiar with the neonatal unit environment and the clinical staff.

Another strategy we adopted was to use opportunistic blood sampling for the infant. An aim of neonatal research is to minimise painful interventions and blood loss. Our approach removed the need for research-specific venepuncture and allowed sampling to be conducted flexibly.

Limitations of study design

Confidential: For Review Only

It was not possible to evaluate if our study design had a direct impact on recruitment rates as recruitment to neonatal studies varies widely depending on the nature of the study and the target population. Enrolment rates of more than 90% were achieved by population-based preterm birth cohorts using traditional study methods such as the EPIPAGE 2 [19], the EPICure 2 [20] and the Express [21]. This suggests that parents of preterm infants are in general willing to participate in birth cohort studies. However, it is recognised that genetic research studies typically experience lower recruitment rates than other types of biomedical research [22]. Our enrolment rate (42.7% of parents approached) is comparable to that reported by the German Neonatal Network, which sought to collect DNA samples from very low birth weight (<1500g) infants at 54 German NICU and successfully enrolled 54% of eligible infants in the first two years [23]. As our study relied entirely on existing neonatal staff, it was possible that recruitment was reduced by competing workloads. Staffing availability and research capacity across the study centres were highly variable. This had a measureable impact on the implementation of the study with approximately one-third of participating neonatal units only initiating recruitment mid-way through the recruitment phase. Despite this, we managed to achieve our recruitment target of 500 families within 11 months, ahead of schedule.

Although our cohort was representative of the national VPT population in neonatal characteristics, selection bias might have been introduced due to the lack of study information materials adapted for non-English speaking families and for those with low literacy. Ethnic and socioeconomic disparities in preterm birth rates are well-described. Future studies should consider how to better integrate study processes targeted at enabling participation of these groups.

Acceptability of research by data linkage

The increasing availability of electronic health records and administrative data provides opportunity for longitudinal cohort follow-up by data linkage. Traditional methods used in neonatal follow-up studies can be time-consuming, expensive and burdensome and are particularly problematic for large sample sizes. In addition, follow-up studies are often compromised by selection bias due to attrition. We found

Confidential: For Review Only

a high level of willingness among parents for their child's routine data to be used in neonatal follow-up research. This corroborates the findings from a systematic review that reported widespread public support for data sharing and linkage for research purposes [24]. In our study, we sought broad consent without specifying the time and type of data that will be accessed. However, Audrey et al revealed the complexity of issues surrounding informed consent for data linkage in a large qualitative study of young people's views [25]. Four hypothetical scenarios were presented to 55 participants to prompt considerations regarding consent requirements. The authors concluded that perceptions were diverse, and sometimes inconsistent. In general, participant views on data linkage were influenced by the social sensitivity of the research question and the likely health benefits in the public interest. Future research in data linkage will benefit from a robust and consistent governance framework that is acceptable to the public.

CONCLUSIONS

We demonstrate the feasibility of a streamlined study approach to achieve a large prospective VPT cohort for clinical and genetic studies and the acceptability of follow-up through data linkage and recall. Our initial cohort is an important resource for studies of genetic risks and preterm birth, and longitudinal health and educational outcomes; our planned larger cohort will amplify this resource. The strategies described provide a template for future cost-effective and efficient cohort development.

Confidential: For Review Only

DECLARATIONS

Acknowledgements

We are grateful to the participating families and staff at all the neonatal units. We thank R Colquhoun, Research Manager and K Ougham, Data Analyst at the Neonatal Data Analysis Unit, Imperial College London for technical and administrative assistance as well as L. Bates, J. Morgan, N. Vinh, A. Evans, L. Hopkins, L. Tram, S. Jaques, S. Lewis at the MRC Centre for Neuropsychiatric Genetics and Genomics, Cardiff University School of Medicine, United Kingdom for laboratory and technical assistance.

Competing interests

NM reports grants from the Medical Research Council, National Institute of Health Research, Department of Health, Westminster Research Fund , Prolacta LIfe Sciences, Chiesi Pharmaceuticals, grants from Shire Pharmaceuticals, HCA International, Healthcare Quality Improvement Partnership, March of Dimes. NM is Director of the Neonatal Data Analysis Unit, Chief Investigator for the National Neonatal Research Database, and Co-Investigator for the Department of Health Funded Maternal and Neonatal Health and Care Policy Research Unit at the University of Oxford. In the last three years NM has served on the Nestle Scientific Advisory Board and the Boards of Trustees of the Royal College of Paediatrics and Child Health, David Harvey Trust, Medical Women's Federation, Medact, Action Cerebral Palsy, and TheirWorld. HSW, LH, MOD and AT declare that they have no competing interests.

Funding

This study was funded by the Medical Research Council, UK (reference MR/N025288/1).

Authors' contributions

NM and AT conceptualized this study; NM, AT and MOD designed the study; AT, MOD, NM and HSW obtained funding. HSW and LH acquired and analysed the data. HSW wrote the first draft of the manuscript. All authors contributed to the editing of the manuscript and have approved the final manuscript.

Confidential: For Review Only

What is already known on this topic

- Infants born very preterm are at increased risk of developing neuropsychiatric conditions such as attention deficit hyperactivity disorder and autism spectrum disorder.
- A large number of genetic risk variants have been identified for neuropsychiatric disorders.

What this study adds

- A large-scale efficient and cost-effective cohort study of preterm infants for genetic investigations in the UK is feasible.
- Longitudinal health and education outcome ascertainment of very preterm infants through routine data linkage is acceptable to parents.

Confidential: For Review Only

REFERENCES

1 Chawanpaiboon S, Vogel JP, Moller A-B, *et al.* Global, regional, and national estimates of levels of preterm birth in 2014: a systematic review and modelling analysis. *Lancet Glob Heal* 2019;**7**:e37–46.

2 Ion RC, Wills AK, Bernal AL. Environmental Tobacco Smoke Exposure in Pregnancy is Associated With Earlier Delivery and Reduced Birth Weight. *Reprod Sci* 2015;**22**:1603–11.

3 Burris HH, Baccarelli AA, Wright RO, *et al.* Epigenetics: linking social and environmental exposures to preterm birth. *Pediatr Res* 2016;**79**:136–40.

4 Strauss JF, Romero R, Gomez-Lopez N, *et al.* Spontaneous preterm birth: advances toward the discovery of genetic predisposition. *Am J Obstet Gynecol* 2018;**218**:294-314.e2.

5 Johnson S, Marlow N. Growing up after extremely preterm birth: Lifespan mental health outcomes. Semin. Fetal Neonatal Med. 2014;**19**:97–104.

6 McCoy BM, Rickert ME, Class QA, *et al.* Mediators of the association between parental severe mental illness and offspring neurodevelopmental problems. *Ann Epidemiol* 2014;**24**:629–34.

7 Venkatesh KK, Riley L, Castro VM, *et al.* Association of Antenatal Depression Symptoms and Antidepressant Treatment With Preterm Birth. *Obstet Gynecol* 2016;**127**:926–33.

8 Walshe M, Rifkin L, Rooney M, *et al.* Psychiatric disorder in young adults born very preterm: role of family history. *Eur Psychiatry* 2008;**23**:527–31.

9 Rogers CE, Lenze SN, Luby JL. Late Preterm Birth, Maternal Depression, and Risk of Preschool Psychiatric Disorders. *J Am Acad Child Adolesc Psychiatry* 2013;**52**:309–18.

10 Cross-Disorder Group of the Psychiatric Genomics Consortium. Identification of risk loci with shared effects on five major psychiatric disorders: a genome-wide analysis. *TLancet* 2013;**381**:1371–9.

11 NHS & Department of Health. Toolkit for high quality neonatal services. London: 2009.

12 German Neonatal Network. https://www.uksh.de/gnn/en/ (accessed 13 Feb2019).

13 Dagle JM, Ryckman KK, Spracklen CN, *et al.* Genetic variants associated with patent ductus arteriosus in extremely preterm infants. *J Perinatol* Published Online First: 5 December 2018.

14 Hartnett ME, Morrison MA, Smith S, *et al.* Genetic Variants Associated With Severe Retinopathy of Prematurity in Extremely Low Birth Weight Infants. *Investig Opthalmology Vis Sci* 2014;**55**:6194.

15 Ment LR, Ådén U, Bauer CR, *et al.* Genes and environment in neonatal intraventricular hemorrhage. *Semin Perinatol* 2015;**39**:592–603.

16 Ambalavanan N, Cotten CM, Page GP, *et al.* Integrated Genomic Analyses in Bronchopulmonary Dysplasia. *J Pediatr* 2015;**166**:531-537.e13.

17 Battersby C, Statnikov Y, Santhakumaran S, *et al.* The United Kingdom National Neonatal Research Database: A validation study. *PLoS One* 2018;**13**:e0201815.

18 Hoehn KS, Nathan A, White LE, *et al.* Parental perception of time and decision-making in neonatal research. *J Perinatol* 2009;**29**:508–11.

19 Ancel P-Y, Goffinet F, EPIPAGE 2 Writing Group. EPIPAGE 2: a preterm birth cohort in France in 2011. *BMC Pediatr* 2014;**14**:97.

Confidential: For Review Only

20 Costeloe KL, Hennessy EM, Haider S, *et al.* Short term outcomes after extreme preterm birth in England: comparison of two birth cohorts in 1995 and 2006 (the EPICure studies). *BMJ* 2012;**345**:e7976–e7976.

21 EXPRESS Group, Fellman V, Hellström-Westas L, *et al.* One-Year Survival of Extremely Preterm Infants After Active Perinatal Care in Sweden. *JAMA* 2009;**301**:2225.

22 Matsui K, Kita Y, Ueshima H. Informed consent, participation in, and withdrawal from a population based cohort study involving genetic analysis. *J Med Ethics* 2005;**31**:385–92.

23 Spiegler J, Stichtenoth G, König IR, *et al.* Health of VLBW infants in Germany at five years of age: What do parents describe? *Early Hum Dev* 2017;**115**:88–92.

24 Aitken M, de St Jorre J, Pagliari C, *et al.* Public responses to the sharing and linkage of health data for research purposes: a systematic review and thematic synthesis of qualitative studies. *BMC Med Ethics* 2016;**17**:73.

25 Audrey S, Brown L, Campbell R, *et al.* Young people's views about consenting to data linkage: findings from the PEARL qualitative study. *BMC Med Res Methodol* 2016;**16**:34.

Confidential: For Review Only

FIGURE TITLES

Figure 1. Flowchart of recruitment and establishment of the cohort for the Study of Preterm Infants and Neurodevelopment Genes, England, United Kingdom, 2017 – 2018

Confidential: For Review Only

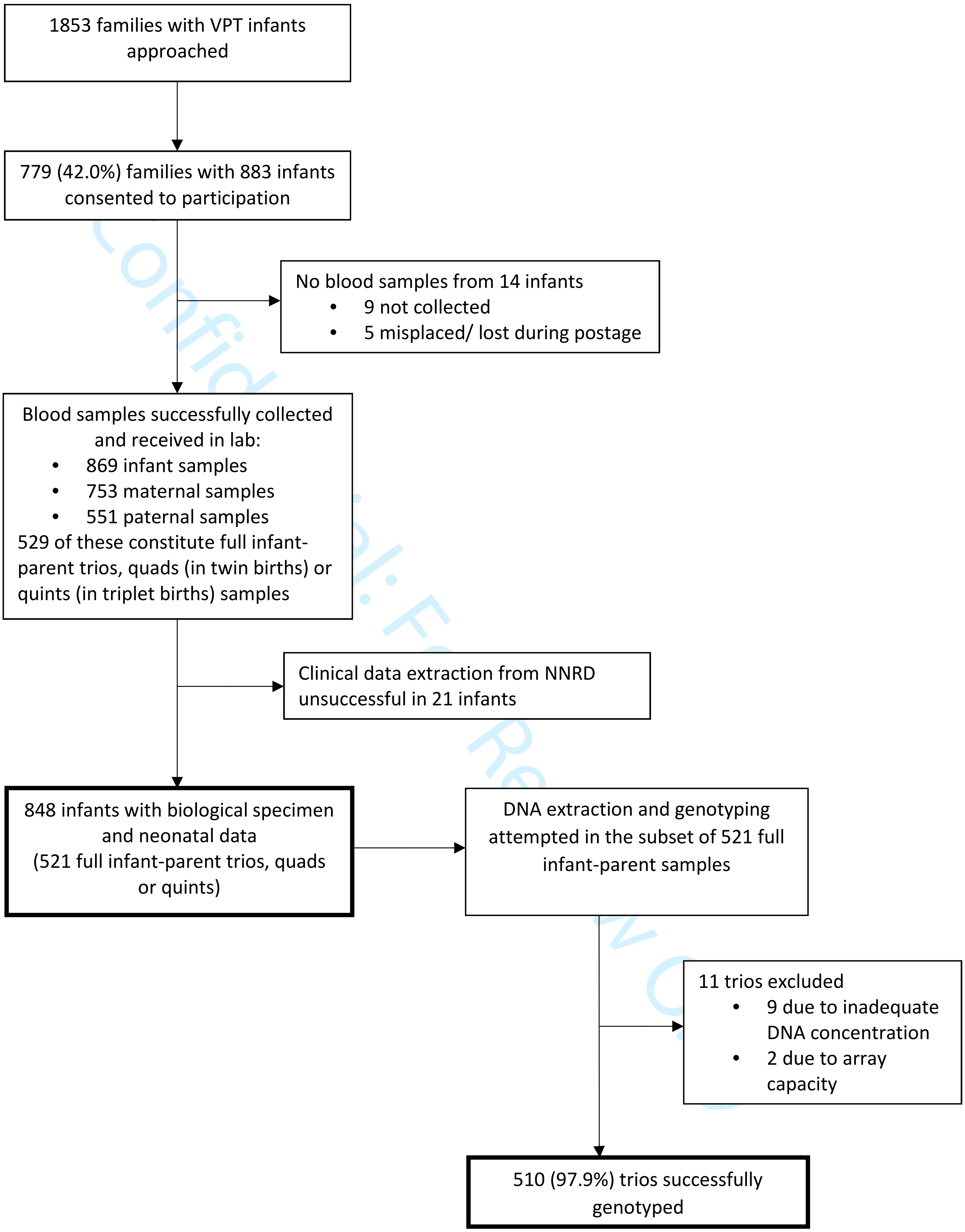
1853 families with VPT infants approached
779 (42.0%) families with 883 infants consented to participation
No blood samples from 14 infants
• 9 not collected
• 5 misplaced/ lost during postage
Blood samples successfully collected and received in lab:
• 869 infant samples
• 753 maternal samples
• 551 paternal samples
529 of these constitute full infant-parent trios, quads (in twin births) or quints (in triplet births) samples
Clinical data extraction from NNRD unsuccessful in 21 infants
848 infants with biological specimen and neonatal data (521 full infant-parent trios, quads or quints)
DNA extraction and genotyping attempted in the subset of 521 full infant-parent samples
11 trios excluded
• 9 due to inadequate DNA concentration
• 2 due to array capacity
510 (97.9%) trios successfully genotyped

Supplementary figure. Geographical distribution of participating neonatal units (each represented by a marker) shown on a map of England, United Kingdom, 2017 – 2018

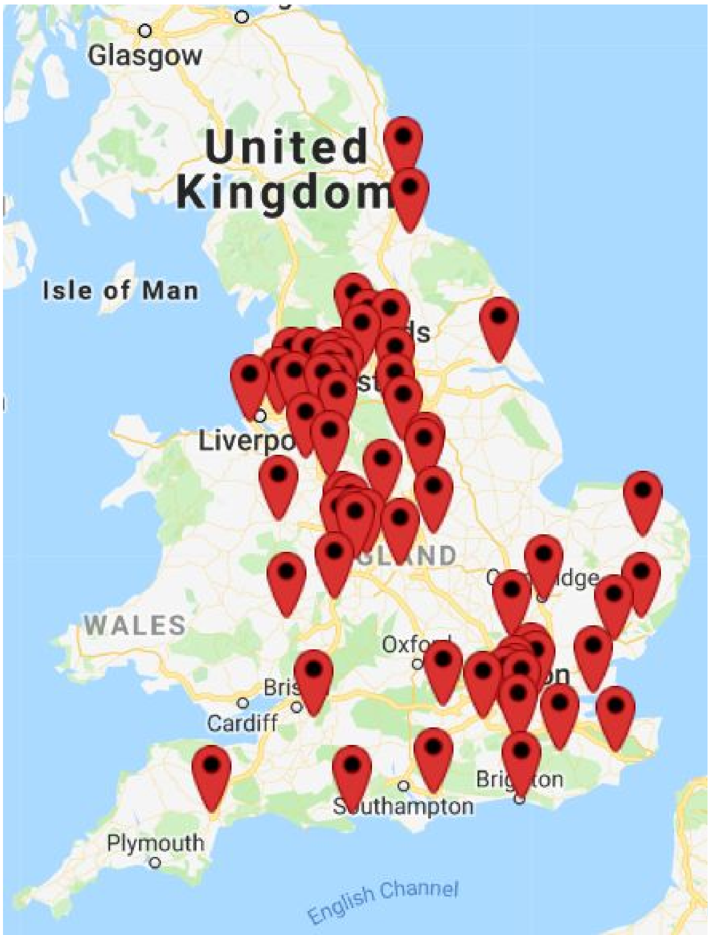

List of participating hospitals:

Airedale General Hospital
Arrowe Park Hospital
Barnsley District General Hospital
Birmingham City Hospital
Birmingham Heartlands Hospital
Birmingham Women's Hospital
Bradford Royal Infirmary
Calderdale Royal Hospital
Chelsea & Westminster Hospital
Chesterfield & North Derbyshire Royal Hospital
Colchester General Hospital
Croydon University Hospital
East Surrey Hospital
Hereford County Hospital
Hull Royal Infirmary
Ipswich Hospital
Kingston Hospital
Leeds Neonatal Service
Leicester General Hospital

For Peer Review Only

Lister Hospital
Macclesfield District General Hospital
Manor Hospital
New Cross Hospital
Newham Hospital
Norfolk & Norwich University Hospital
North Manchester
North Tyneside General Hospital
Nottingham City Hospital/
Poole General Hospital
Queen Alexandra Hospital (Portsmouth)
Queen's Hospital, Burton on Trent
Queen's Medical Centre, Nottingham University Hospital
Rosie Maternity Hospital, Addenbrookes
Royal Albert Edward Infirmary
Royal Berkshire Hospital
Royal Bolton Hospital
Royal Devon & Exeter Hospital
Royal Oldham Hospital
Royal Shrewsbury Hospital
Royal Stoke University Hospital
Royal Sussex County Hospital
Royal United Hospital, Bath
Russells Hall Hospital
Southend Hospital
St George's Hospital
St Mary's Hospital, Manchester
St Peter's Hospital
Stepping Hill Hospital
Sunderland Royal Hospital
Tameside General Hospital
The Jessop Wing, Sheffield
Tunbridge Wells Hospital
University Hospital Coventry
University Hospital of North Tees
University Hospital of South Manchester
Warrington Hospital
Whipps Cross University Hospital/
Whiston Hospital
William Harvey Hospital
Worcestershire Royal Hospital

Confidential: For Review Only

BMJ Paediatrics Open

Pilot study to establish a prospective neonatal cohort: Study of Preterm Infants and Neurodevelopmental Genes (SPRING)

Journal:	*BMJ Paediatrics Open*
Manuscript ID	bmjpo-2020-000648.R1
Article Type:	Original research
Date Submitted by the Author:	30-Apr-2020

Complete List of Authors:	Wong, Hilary; University of Cambridge School of Clinical Medicine, Department of Paediatrics<br>Hopkins, Lucinda; Cardiff University, MRC Centre for Neuropsychiatric Genetics and Genomics<br>O'Donovan, Michael; Cardiff University, MRC Centre for Neuropsychiatric Genetics and Genomics<br>Thapar, Anita; Cardiff University, MRC Centre for Neuropsychiatric Genetics and Genomics<br>Modi, Neena; Imperial College London Department of Medicine, Section of Neonatal Medicine

Keywords:	Autism, Epidemiology, Genetics, Neonatology, Neurodevelopment

SCHOLARONE™
Manuscripts

I, the Submitting Author has the right to grant and does grant on behalf of all authors of the Work (as defined in the below author licence), an exclusive licence and/or a non-exclusive licence for contributions from authors who are: i) UK Crown employees; ii) where BMJ has agreed a CC-BY licence shall apply, and/or iii) in accordance with the terms applicable for US Federal Government officers or employees acting as part of their official duties; on a worldwide, perpetual, irrevocable, royalty-free basis to BMJ Publishing Group Ltd ("BMJ") its licensees and where the relevant Journal is co-owned by BMJ to the co-owners of the Journal, to publish the Work in this journal and any other BMJ products and to exploit all rights, as set out in our licence.

The Submitting Author accepts and understands that any supply made under these terms is made by BMJ to the Submitting Author unless you are acting as an employee on behalf of your employer or a postgraduate student of an affiliated institution which is paying any applicable article publishing charge ("APC") for Open Access articles. Where the Submitting Author wishes to make the Work available on an Open Access basis (and intends to pay the relevant APC), the terms of reuse of such Open Access shall be governed by a Creative Commons licence – details of these licences and which Creative Commons licence will apply to this Work are set out in our licence referred to above.

Other than as permitted in any relevant BMJ Author's Self Archiving Policies, I confirm this Work has not been accepted for publication elsewhere, is not being considered for publication elsewhere and does not duplicate material already published. I confirm all authors consent to publication of this Work and authorise the granting of this licence.

Confidential: For Review Only

Pilot study to establish a prospective neonatal cohort: Study of Preterm Infants and Neurodevelopmental Genes (SPRING)

Authors:

Hilary S Wong[1]

Lucinda Hopkins[2]

Michael C O'Donovan[2]

Anita Thapar[2]

Neena Modi[3]

Affiliations:

1. Department of Paediatrics, University of Cambridge, Cambridge, United Kingdom
2. MRC Centre for Neuropsychiatric Genetics and Genomics, Cardiff University School of Medicine, Cardiff, United Kingdom
3. Section of Neonatal Medicine, Chelsea and Westminster campus, Imperial College London, London, United Kingdom

Corresponding author:

Professor Neena Modi
Section of Neonatal Medicine
Imperial College London
Chelsea and Westminster campus
369 Fulham Road
London SW10 9NH
Email: n.modi@imperial.ac.uk
Telephone: 020 3315 5101

Word count: 2801 words

Confidential: For Review Only

ABSTRACT

Background: Genetic risk variants and preterm birth are early and potent risk factors for later neuropsychiatric disorders. To understand the interrelationships between these factors, a large-scale genetic study of very preterm (VPT, <32 weeks gestation) infants with prospective follow-up is required. In this paper we describe a streamlined study approach, using efficient processes for biological and clinical data collection, to feasibly establish such a cohort.

Methods: We sought to recruit 500 VPT families within a one-year period from neonatal units. Treating clinical teams recruited eligible participants, obtained parent consent, collected blood samples and posted specimens to the research laboratory. We extracted all clinical data from the National Neonatal Research Database, an existing UK resource that captures daily patient-level data on all VPT infants.

Results: Between May 2017 and June 2018, we established a cohort of 848 VPT infants and their parents from 60 English neonatal units. The study population (median (inter-quartile range) gestation 28.9 (26-30) weeks; birthweight 1120 (886-1420) grams) represented 18.9% of eligible infants born at the study sites during the recruitment period (n= 4491). From the subset of 521 complete family trios, we successfully completed genotyping for 510 (97.9%) trios. Of the original 883 infants whose parents consented to participate, the parents of 796 (90.1%) infants agreed to future data linkage and 794 (89.9%) agreed to be recalled.

Conclusion: We demonstrate the feasibility and acceptability of streamlined strategies for genetic, neonatal and longitudinal data collection and provide a template for future cost-effective and efficient cohort development.

(248 of max 250 words)

Keywords: Preterm birth, prospective cohort study, neurodevelopment, autism spectrum disorder, attention deficit hyperactivity disorder, genetics, epidemiology, data linkage

https://mc.manuscriptcentral.com/bmjpo

Confidential: For Review Only

BACKGROUND

Globally, approximately 15 million infants are born preterm (<37 weeks gestation) each year and the prevalence is rising [1]. The causes of spontaneous preterm births and the conditions justifying 'medically-indicated' preterm birth are complex and poorly understood, with both environmental factors [2,3] and genetic components [4] implicated. Preterm infants experience significant adversities and are at elevated risk of intellectual disability, behavioural and mental health problems, with approximately a quarter of children born extremely preterm meeting neuropsychiatric diagnostic criteria by childhood [5]. However, there is considerable heterogeneity in outcomes, the causes of which are unknown.

Previous epidemiological studies have suggested family and genetic factors may contribute to the link between preterm birth and later neuropsychiatric disorders. Many of these studies have observed an association between maternal mental health problems and risk of preterm birth [6,7]. Positive family history of psychiatric disorder further contributes to the risk of adverse mental health outcomes among individuals born preterm [8,9]. Genomic studies have identified large numbers of genetic risk variants for major neuropsychiatric disorders, including those for attention deficit hyperactivity disorder and autism spectrum disorder, which are the neuropsychiatric disorders most strongly associated with preterm birth in childhood [10]. However, the relevance of these genetic risk variants for those born preterm has not been investigated.

A large preterm birth cohort with genomic data and prospective clinical phenotyping would be required to investigate the relationship between genetic risks, preterm birth and neuropsychiatric outcomes. There is no sufficiently powered existing prospective cohort of preterm infants for such investigations and the costs, time and resource requirements for recruitment and data collection by established methods are likely to be prohibitive. Our aims were to examine the feasibility of large-scale recruitment and DNA collection in a multi-centre setting, by capitalising on existing research infrastructure in the UK to streamline operational efficiencies, and the acceptability to parents of prospective follow-up through

https://mc.manuscriptcentral.com/bmjpo

routine data linkage and direct recall for detailed phenotyping. Our secondary aim was to conduct a preliminary investigation to explore the hypothesis that the very preterm (VPT, <32 weeks gestation) population is enriched for rare pathogenic copy number variants; the results of this investigation were reported in a separate paper [11].

Confidential: For Review Only

METHODS

Study sites and recruitment

We invited expressions of interest in participation as study sites from all 43 neonatal intensive care units (NICU) and 77 local neonatal units (LNU) in England. In the UK, neonatal care is provided within managed clinical networks where LNU provide the full range of care, including short periods of intensive care, for babies delivered in the nominal catchment area of the hospital [12]. Most babies born over 27 weeks gestation will usually receive their full care in their LNU. Babies who are more preterm or those who require complex or longer-term intensive care are transferred to a NICU, where tertiary specialist care is provided. From the units that expressed interest, we selected study sites based on the potential number of eligible participants, geographical coverage and record of accomplishment of research activities in the unit.. The study was included in the National Institute for Health Research Clinical Research Network Portfolio, which allowed us to access support, such as CRN-funded research nurses in England. Being a 'portfolio study' also acts as an incentive for recruitment activities from neonatal units as the accrual data are used to inform future allocations of NHS research support funding to hospital trusts. All study procedures were carried out by the treating clinical or research staff based locally at the participating study sites. These local staff received training in the study procedures from the investigator team using training videos and teleconferences.

Recruitment took place between 1 May 2017 and 30 June 2018. Eligible participants were infants born VPT and their parents. Whilst we aimed to recruit both parents for trio-based genome analysis, single-

parent families were also included. Similarly, we recruited infants where one or both parents declined to participate in the study themselves. We did not recruit parents known to be genetically unrelated to the child, for instance if the pregnancy had arisen from a donor gamete. Potential participants could be approached and recruited at any point during their neonatal hospitalisation at the discretion of the local research staff. Informed written consent was obtained from each parent (for themselves) and their offspring at the local site. We sought to recruit 500 VPT infants over the one-year recruitment phase.

Biological specimen collection and laboratory processing

Blood samples from participants were collected into EDTA specimen bottles. For infants, 0.5 to 1ml of blood were collected by heel lance, venepuncture or from an indwelling arterial catheter during blood sampling for routine clinical care to avoid an additional intervention for the infant. If the target volume was not achieved, a second aliquot was obtained, also collected during routine sampling. Five ml of blood was collected from each parent. The blood specimens were labelled using barcodes and a unique participant study number, packaged in a designated mailing box for biological specimens, in accordance with UN3373 regulations and P650 packaging instructions, and posted to the research laboratory at the MRC Centre for Neuropsychiatric Genetics and Genomics (MRC CNGG), Cardiff University within 7 days of sample collection using UK standard postal services. No laboratory processing of the samples took place at the recruitment study sites.

DNA was extracted from the blood specimens in-house at the MRC CNGG, from full sets of parent-infant trios in the first instance, using GE Healthcare DNA extraction kits. DNA sample quantification was determined using Quant-iT PicoGreen dsDNA assay kits and samples were genotyped using Illumina OmniExpress BeadChip arrays. Samples with concentrations below 50ng/ul were excluded from genotyping. DNA was considered of adequate quality on successful completion of array based genotyping.

Clinical data collection

Confidential: For Review Only

To minimise research workload and participant burden, we capitalised on the existing National Neonatal Research Database (NNRD) to access relevant clinical data on the research participants. Investigator NM led the establishment of the NNRD in 2007 to support the use of clinical data to improve neonatal care and outcomes, and the UK Neonatal Collaborative comprising all neonatal units that contributed data. Patient-level point-of-care electronic data entered by treating clinicians on all infants admitted to the neonatal units are released with permission from the Caldicott Guardians to the Neonatal Data Analysis Unit at Imperial College London, where they are merged and cleaned to create the NNRD (https://www.imperial.ac.uk/neonatal-data-analysis-unit). Data captured on the NNRD includes demographic details, daily records of intervention and treatments through the neonatal inpatient stay, diagnoses and follow-up health status at age two years. The NNRD has complete population coverage of all neonatal unit admissions, including all VPT births in England, Wales and Scotland (approximately 7000 annually) and is approved by the UK National Research Ethics Service (10/80803/151) as a research database and by the Health Research Authority Confidentiality Advisory Group (8-05(f)/0210).

Upon receipt of parent signed authorisation, the unique NNRD identifier for each participating infant was obtained from his/her neonatologist. All relevant participant clinical data, including gestational age, birth weight, sex, time of onset of labour and maternal postcode, was extracted from the NNRD.

Consent for follow-up

Permissions were sought from parents of participating infants to conduct follow-up studies by means of linkage of the genetic and clinical data to future routine health and educational records, in order to reduce the research burden associated with long-term follow-up and outcome ascertainment. This was indicated by the parents' response to the following statement in the consent form: "*I give permission for the research team to link the study information on my baby to records that the NHS and other public organisations hold*". Consent was also sought for re-contacting the family in the future for detailed phenotyping through face-to-face or questionnaire-based assessments.Parents were made aware of their right to withdraw from the study at any point by contacting the researchers.

https://mc.manuscriptcentral.com/bmjpo

Research ethics

This study was reviewed and approved by the Heath Research Authority and the National Health Service Research Ethics Committee (reference 16/WA/0324).

Patient involvement

We involved Bliss, the leading national preterm charity in the design and funding application for the study. We consulted parent representatives in developing the study materials including the participant information sheet. They will be consulted for the public dissemination of the findings from this research.

RESULTS

Recruitment and establishment of study population

We received expressions of interest from 75 out of 120 neonatal units in England. Of these, we selected 60 neonatal units (24 NICU and 36 LNU) to be study sites. These provided an extensive population catchment coverage in England (see supplementary material for geographical coverage and list of participating hospitals).

During the 14-month recruitment period, 1853 families were approached and 779 (42.0%) families, consisting of 883 infants, consented to participation (figure 1). Blood samples were not collected in 9 infants prior to their discharge. In 5 cases, the sample was lost during postage. Blood samples from 869 infants, 753 mothers and 551 fathers were successfully received in the MRC CNGG laboratory. Of these samples, there were 529 full infant-parent trios, quads (in twin births) or quints (in triplet births).

Clinical data extraction from the NNRD was successful in 848 (97.6%) infants whose blood samples had arrived at the laboratory. The demographic and neonatal characteristics of these participants are summarised in table 1.

During the recruitment period, a total of 8998 infants were born at <32 weeks gestation in England, with 4491 of whom being born at participating study sites. Therefore, this study cohort represent 18.9% of

https://mc.manuscriptcentral.com/bmjpo

Confidential: For Review Only

eligible infants born at study sites and 9.4% of the national VPT population. Our study sample was representative of the national VPT population in gestational age, birth weight, proportion of boys, singleton pregnancies, spontaneous onset of preterm labour and mode of delivery (table 1).

Table 1 Neonatal characteristics of SPRING participants and of all VPT infants born in the participating study sites and in England during the recruitment period (May 2017 - June 2018)

	**SPRING participants** (n=848)	**All eligible infants born in study sites** (n=4491)	**National cohort** (n=8998)
Gestational age in weeks median (IQR)	28.9 (26 – 30)	29 (27 – 30)	29 (27 – 30)
Birth weight in grams median (IQR)	1120 (886 – 1420)	1175 (882 – 1480)	1170 (880 – 1470)
Boys, n (%)	456 (53.8)	2469 (55.0)	4900 (54.5)
Singleton pregnancy, n (%)	638 (75.2)	3359 (74.8)	6706 (74.5)
Spontaneous onset of labour, n (%)	474 (55.9)	2431 (54.1)	4815 (53.5)
Caesearean section delivery, n (%)	498 (58.7)	2584 (57.5)	5087 (56.5)
Maternal age at birth in years, Median (IQR)	31 (27-35)	Information not available	Information not available

DNA extraction and quality

DNA was extracted from a subset of blood samples from 521 full parent-infant trios who had successful linkage to infant clinical data. Of these, nine trios were excluded from genotyping due to inadequate DNA sample concentration (below 50ng/ul) of one of the trio members. An additional two trios were excluded due to array capacity, resulting in 510 trios (97.9%) of this subset being successfully genotyped.

Follow-up

Of the 883 infants whose parents consented to participate in the study, 796 (90.1%) agreed for their child's future health and educational data to be linked to the research data. Consent was also obtained from the parents of 794 (89.9%) for future direct contact.

Confidential: For Review Only

DISCUSSION

Large-scale prospective cohort studies are time-consuming and expensive to conduct. Cohorts of special patient or high-risk groups, such as preterm infants, require assembly from multiple centres across large geographical areas, which poses additional logistical challenge. In our pilot study, we overcame some of these barriers by optimising research operational efficiencies. Although other large-scale preterm neonatal cohorts with genetic data exist internationally [13–17], none have collected parent DNA samples for trio-based genetic analyses. Parent blood samples for trio-based analysis is essential to identify likely pathogenic *de novo* variants. Our study approaches were purposefully designed to reduce research burden, respectful of the needs of preterm neonates and their families and sensitive to the culture within neonatal units. Within one year, we successfully established a large contemporaneous VPT neonatal cohort for genetic investigations.

Choice of recruitment and data collection methods

The completeness and quality of data held in the NNRD has been validated for use in research [18]. The advantages of leveraging routine electronic health records for research are well-recognised. These include less intrusive data collection, elimination of the need for duplicate data entry, and reduced research burden and cost. The reduced demands allowed us to incorporate the study activities within the workload of existing clinical and research staff at the participating centres, thereby minimising research costs.

As no research-specific prospective data collection was required, our study design also removed the time pressure to recruit newborn infants immediately after birth, particularly as this may be a stressful and emotional time for parents. Time constraints upon decision-making reduces willingness to participate [19]. Participants in our study were usually recruited a few weeks after delivery, when the parents had become more familiar with the neonatal unit environment and the clinical staff.

Confidential: For Review Only

Another strategy we adopted was to use opportunistic blood sampling for the infant. An aim of neonatal research is to minimise painful interventions and blood loss. Our approach removed the need for research-specific venepuncture and allowed sampling to be conducted flexibly.

Limitations of study design

It was not possible to evaluate if our study design had a direct impact on recruitment rates as recruitment to neonatal studies varies widely depending on the nature of the study and the target population. Enrolment rates of more than 90% were achieved by population-based preterm birth cohorts using traditional study methods such as the EPIPAGE 2 [20], the EPICure 2 [21] and the Express [22]. This suggests that parents of preterm infants are in general willing to participate in birth cohort studies. However, it is recognised that genetic research studies typically experience lower recruitment rates than other types of biomedical research [23]. Our enrolment rate (42.7% of parents approached) is comparable to that reported by the German Neonatal Network, which sought to collect DNA samples from very low birth weight (<1500g) infants at 54 German NICU and successfully enrolled 54% of eligible infants in the first two years [24]. As our study relied entirely on existing neonatal staff, it was possible that recruitment was reduced by competing workloads. Staffing availability and research capacity across the study centres were highly variable. This had a measureable impact on the implementation of the study with approximately one-third of participating neonatal units only initiating recruitment mid-way through the recruitment phase. Despite this, we managed to achieve our recruitment target of 500 families within 11 months, ahead of schedule. We did not include the recruitment of a 'term control group', which will be vital for direct case-control comparison, in this pilot study. The recruitment of well, term-born born infants and their parents for genetic analyses will undoubtedly lend new logistical and ethical challenges that need further exploration.

Although our cohort was representative of the national VPT population in neonatal characteristics, selection bias might have been introduced due to the lack of study information materials adapted for non-English speaking families and for those with low literacy. Ethnic and socioeconomic disparities in

https://mc.manuscriptcentral.com/bmjpo

preterm birth rates are well-described. Future studies should consider how to better integrate study processes targeted at enabling participation of these groups.

Although the NNRD has been successfully utilized for clinical research in the UK, the utility of the database for the study of environmental influences and outcome phenotyping in the context of genetic investigations has yet to be demonstrated, and will need to be examined in future studies. We had not allowed for the collection of different biological tissues at different time points to enable epigenetic analyses, as this would have added onto the research burden of the clinical teams and the families participating. The feasibility and acceptability of using archived or surplus biological specimens collected during routine clinical care e.g. archived newborn blood spot for this purpose could be further explored.

Acceptability of research by data linkage

The increasing availability of electronic health records and administrative data provides opportunity for longitudinal cohort follow-up by data linkage. Traditional methods used in neonatal follow-up studies can be time-consuming, expensive and burdensome and are particularly problematic for large sample sizes. In addition, follow-up studies are often compromised by selection bias due to attrition. We found a high level of willingness among parents for their child's routine data to be used in neonatal follow-up research. This corroborates the findings from a systematic review that reported widespread public support for data sharing and linkage for research purposes [25]. In our study, we sought broad consent without specifying the time and type of data that will be accessed. However, Audrey et al revealed the complexity of issues surrounding informed consent for data linkage in a large qualitative study of young people's views [26]. Four hypothetical scenarios were presented to 55 participants to prompt considerations regarding consent requirements. The authors concluded that perceptions were diverse, and sometimes inconsistent. In general, participant views on data linkage were influenced by the social sensitivity of the research question and the likely health benefits in the public interest. Future research in data linkage will benefit from a robust and consistent governance framework that is acceptable to the public.

Confidential: For Review Only

CONCLUSIONS

We demonstrate the feasibility of a streamlined study approach to achieve a large prospective VPT cohort for clinical and genetic studies and the acceptability of follow-up through data linkage and recall. Our initial cohort is an important resource for studies of genetic risks and preterm birth, and longitudinal health and educational outcomes; our planned larger cohort will amplify this resource. The strategies described provide a template for future cost-effective and efficient cohort development.

Confidential: For Review Only

DECLARATIONS

Acknowledgements

We are grateful to the participating families and staff at all the neonatal units. We thank R Colquhoun, Research Manager and K Ougham, Data Analyst at the Neonatal Data Analysis Unit, Imperial College London for technical and administrative assistance as well as L. Bates, J. Morgan, N. Vinh, A. Evans, L. Hopkins, L. Tram, S. Jaques, S. Lewis at the MRC Centre for Neuropsychiatric Genetics and Genomics, Cardiff University School of Medicine, United Kingdom for laboratory and technical assistance.

Competing interests

NM reports grants from the Medical Research Council, National Institute of Health Research, Department of Health, Westminster Research Fund , Prolacta LIfe Sciences, Chiesi Pharmaceuticals, grants from Shire Pharmaceuticals, HCA International, Healthcare Quality Improvement Partnership, March of Dimes. NM is Director of the Neonatal Data Analysis Unit, Chief Investigator for the National Neonatal Research Database, and Co-Investigator for the Department of Health Funded Maternal and Neonatal Health and Care Policy Research Unit at the University of Oxford. In the last three years NM has served on the Nestle Scientific Advisory Board and the Boards of Trustees of the Royal College of Paediatrics and Child Health, David Harvey Trust, Medical Women's Federation, Medact, Action Cerebral Palsy, and TheirWorld. HSW, LH, MOD and AT declare that they have no competing interests.

Funding

This study was funded by the Medical Research Council, UK (reference MR/N025288/1).

Authors' contributions

NM and AT conceptualized this study; NM, AT and MOD designed the study; AT, MOD, NM and HSW obtained funding. HSW and LH acquired and analysed the data. HSW wrote the first draft of the manuscript. All authors contributed to the editing of the manuscript and have approved the final manuscript.

Confidential: For Review Only

What is already known on this topic

- Infants born very preterm are at increased risk of developing neuropsychiatric conditions such as attention deficit hyperactivity disorder and autism spectrum disorder.
- A large number of genetic risk variants have been identified for neuropsychiatric disorders.

What this study adds

- A large-scale efficient and cost-effective cohort study of preterm infants for genetic investigations in the UK is feasible.
- Longitudinal health and education outcome ascertainment of very preterm infants through routine data linkage is acceptable to parents.

Confidential: For Review Only

REFERENCES

1 Chawanpaiboon S, Vogel JP, Moller A-B, *et al.* Global, regional, and national estimates of levels of preterm birth in 2014: a systematic review and modelling analysis. *Lancet Glob Heal* 2019;**7**:e37–46.

2 Ion RC, Wills AK, Bernal AL. Environmental Tobacco Smoke Exposure in Pregnancy is Associated With Earlier Delivery and Reduced Birth Weight. *Reprod Sci* 2015;**22**:1603–11.

3 Burris HH, Baccarelli AA, Wright RO, *et al.* Epigenetics: linking social and environmental exposures to preterm birth. *Pediatr Res* 2016;**79**:136–40.

4 Strauss JF, Romero R, Gomez-Lopez N, *et al.* Spontaneous preterm birth: advances toward the discovery of genetic predisposition. *Am J Obstet Gynecol* 2018;**218**:294-314.e2.

5 Johnson S, Marlow N. Growing up after extremely preterm birth: Lifespan mental health outcomes. *Semin Fetal Neonatal Med* 2014;**19**:97–104.

6 McCoy BM, Rickert ME, Class QA, *et al.* Mediators of the association between parental severe mental illness and offspring neurodevelopmental problems. *Ann Epidemiol* 2014;**24**:629–34.

7 Venkatesh KK, Riley L, Castro VM, *et al.* Association of Antenatal Depression Symptoms and Antidepressant Treatment With Preterm Birth. *Obstet Gynecol* 2016;**127**:926–33.

8 Walshe M, Rifkin L, Rooney M, *et al.* Psychiatric disorder in young adults born very preterm: role of family history. *Eur Psychiatry* 2008;**23**:527–31.

9 Rogers CE, Lenze SN, Luby JL. Late Preterm Birth, Maternal Depression, and Risk of Preschool Psychiatric Disorders. *J Am Acad Child Adolesc Psychiatry* 2013;**52**:309–18.

10 Cross-Disorder Group of the Psychiatric Genomics Consortium. Identification of risk loci with shared effects on five major psychiatric disorders: a genome-wide analysis. *TLancet* 2013;**381**:1371–9.

11 Wong HS, Wadon M, Evans A, *et al.* Contribution of de novo and inherited rare CNVs to very preterm birth. *J Med Genet* [Published Online First]. https://jmg.bmj.com/content/early/2020/02/12/jmedgenet-2019-106619.long (accessed 30 April 2020)

12 NHS & Department of Health. Toolkit for high quality neonatal services. London: 2009.

13 German Neonatal Network. https://www.uksh.de/gnn/en/ (accessed 13 Feb 2019).

14 Dagle JM, Ryckman KK, Spracklen CN, *et al.* Genetic variants associated with patent ductus arteriosus in extremely preterm infants. *J Perinatol* 2019;**39**:401-8.

15 Hartnett ME, Morrison MA, Smith S, *et al.* Genetic Variants Associated With Severe Retinopathy of Prematurity in Extremely Low Birth Weight Infants. *Investig Opthalmology Vis Sci* 2014;**55**:6194.

16 Ment LR, Ådén U, Bauer CR, *et al.* Genes and environment in neonatal intraventricular hemorrhage. *Semin Perinatol* 2015;**39**:592–603.

17 Ambalavanan N, Cotten CM, Page GP, *et al.* Integrated Genomic Analyses in Bronchopulmonary Dysplasia. *J Pediatr* 2015;**166**:531-537.e13.

18 Battersby C, Statnikov Y, Santhakumaran S, *et al.* The United Kingdom National Neonatal Research Database: A validation study. *PLoS One* 2018;**13**:e0201815.

Confidential: For Review Only

19 Hoehn KS, Nathan A, White LE, *et al.* Parental perception of time and decision-making in neonatal research. *J Perinatol* 2009;**29**:508–11.

20 Ancel P-Y, Goffinet F, EPIPAGE 2 Writing Group. EPIPAGE 2: a preterm birth cohort in France in 2011. *BMC Pediatr* 2014;**14**:97.

21 Costeloe KL, Hennessy EM, Haider S, *et al.* Short term outcomes after extreme preterm birth in England: comparison of two birth cohorts in 1995 and 2006 (the EPICure studies). *BMJ* 2012;**345**:e7976–e7976.

22 EXPRESS Group, Fellman V, Hellström-Westas L, *et al.* One-Year Survival of Extremely Preterm Infants After Active Perinatal Care in Sweden. *JAMA* 2009;**301**:2225.

23 Matsui K, Kita Y, Ueshima H. Informed consent, participation in, and withdrawal from a population based cohort study involving genetic analysis. *J Med Ethics* 2005;**31**:385–92.

24 Spiegler J, Stichtenoth G, König IR, *et al.* Health of VLBW infants in Germany at five years of age: What do parents describe? *Early Hum Dev* 2017;**115**:88–92.

25 Aitken M, de St Jorre J, Pagliari C, *et al.* Public responses to the sharing and linkage of health data for research purposes: a systematic review and thematic synthesis of qualitative studies. *BMC Med Ethics* 2016;**17**:73.

26 Audrey S, Brown L, Campbell R, *et al.* Young people's views about consenting to data linkage: findings from the PEARL qualitative study. *BMC Med Res Methodol* 2016;**16**:34.

Confidential: For Review Only

FIGURE TITLES

Figure 1. Flowchart of recruitment and establishment of the cohort for the Study of Preterm Infants and Neurodevelopment Genes, England, United Kingdom, 2017 – 2018

Confidential: For Review Only

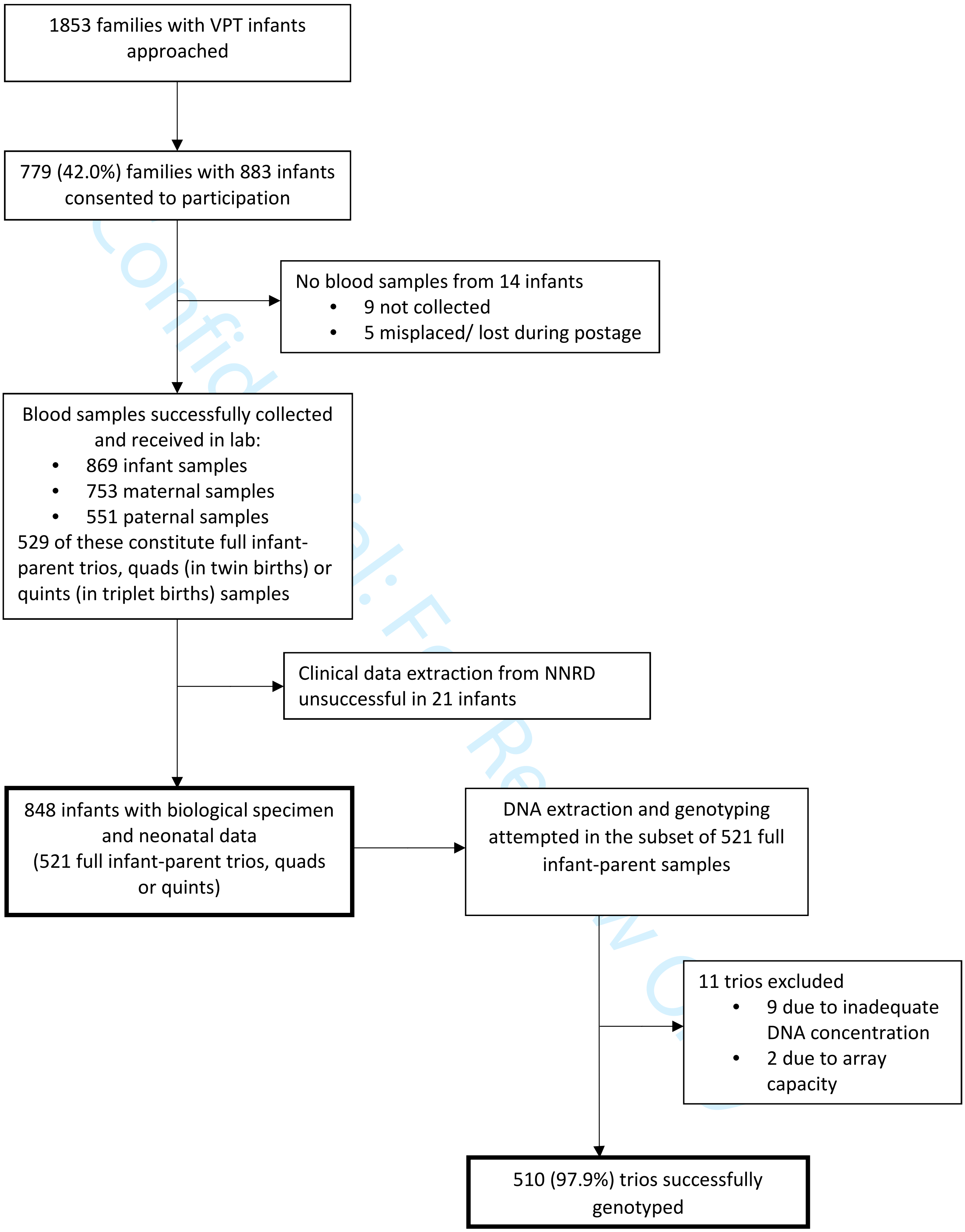
1853 families with VPT infants approached
779 (42.0%) families with 883 infants consented to participation
No blood samples from 14 infants
• 9 not collected
• 5 misplaced/ lost during postage
Blood samples successfully collected and received in lab:
• 869 infant samples
• 753 maternal samples
• 551 paternal samples
529 of these constitute full infant-parent trios, quads (in twin births) or quints (in triplet births) samples
Clinical data extraction from NNRD unsuccessful in 21 infants
848 infants with biological specimen and neonatal data (521 full infant-parent trios, quads or quints)
DNA extraction and genotyping attempted in the subset of 521 full infant-parent samples
11 trios excluded
• 9 due to inadequate DNA concentration
• 2 due to array capacity
510 (97.9%) trios successfully genotyped

Supplementary figure. Geographical distribution of participating neonatal units (each represented by a marker) shown on a map of England, United Kingdom, 2017 – 2018

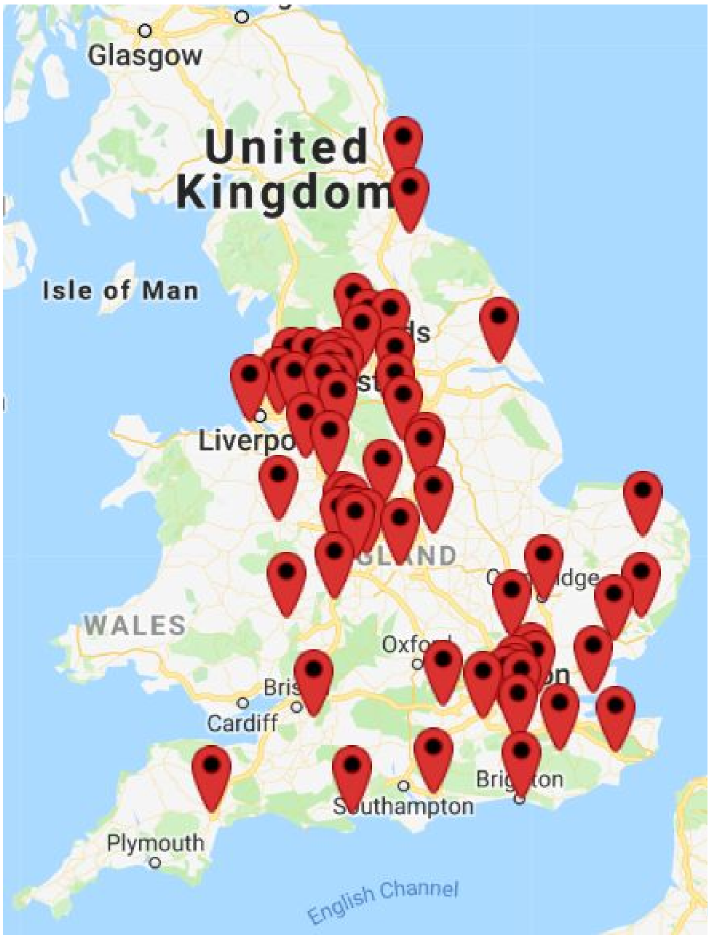

<u>**List of participating hospitals**</u>:

Airedale General Hospital
Arrowe Park Hospital
Barnsley District General Hospital
Birmingham City Hospital
Birmingham Heartlands Hospital
Birmingham Women's Hospital
Bradford Royal Infirmary
Calderdale Royal Hospital
Chelsea & Westminster Hospital
Chesterfield & North Derbyshire Royal Hospital
Colchester General Hospital
Croydon University Hospital
East Surrey Hospital
Hereford County Hospital
Hull Royal Infirmary
Ipswich Hospital
Kingston Hospital
Leeds Neonatal Service
Leicester General Hospital

Review Only

Lister Hospital
Macclesfield District General Hospital
Manor Hospital
New Cross Hospital
Newham Hospital
Norfolk & Norwich University Hospital
North Manchester
North Tyneside General Hospital
Nottingham City Hospital/
Poole General Hospital
Queen Alexandra Hospital (Portsmouth)
Queen's Hospital, Burton on Trent
Queen's Medical Centre, Nottingham University Hospital
Rosie Maternity Hospital, Addenbrookes
Royal Albert Edward Infirmary
Royal Berkshire Hospital
Royal Bolton Hospital
Royal Devon & Exeter Hospital
Royal Oldham Hospital
Royal Shrewsbury Hospital
Royal Stoke University Hospital
Royal Sussex County Hospital
Royal United Hospital, Bath
Russells Hall Hospital
Southend Hospital
St George's Hospital
St Mary's Hospital, Manchester
St Peter's Hospital
Stepping Hill Hospital
Sunderland Royal Hospital
Tameside General Hospital
The Jessop Wing, Sheffield
Tunbridge Wells Hospital
University Hospital Coventry
University Hospital of North Tees
University Hospital of South Manchester
Warrington Hospital
Whipps Cross University Hospital/
Whiston Hospital
William Harvey Hospital
Worcestershire Royal Hospital

Confidential: For Review Only
